# Comparison between pystan and numpyro in Bayesian item response theory: evaluation of agreement of estimated latent parameters and sampling performance

**DOI:** 10.7717/peerj-cs.1620

**Published:** 2023-10-05

**Authors:** Mizuho Nishio, Eiji Ota, Hidetoshi Matsuo, Takaaki Matsunaga, Aki Miyazaki, Takamichi Murakami

**Affiliations:** 1 Department of Radiology, Kobe University Graduate School of Medicine, Kobe, Japan; 2 Futaba Numerical Technologies, Iruma, Japan

**Keywords:** Item response theory, Markov chain Monte Carlo, Graphics processing unit

## Abstract

**Purpose:**

The purpose of this study is to compare two libraries dedicated to the Markov chain Monte Carlo method: pystan and numpyro. In the comparison, we mainly focused on the agreement of estimated latent parameters and the performance of sampling using the Markov chain Monte Carlo method in Bayesian item response theory (IRT).

**Materials and methods:**

Bayesian 1PL-IRT and 2PL-IRT were implemented with pystan and numpyro. Then, the Bayesian 1PL-IRT and 2PL-IRT were applied to two types of medical data obtained from a published article. The same prior distributions of latent parameters were used in both pystan and numpyro. Estimation results of latent parameters of 1PL-IRT and 2PL-IRT were compared between pystan and numpyro. Additionally, the computational cost of the Markov chain Monte Carlo method was compared between the two libraries. To evaluate the computational cost of IRT models, simulation data were generated from the medical data and numpyro.

**Results:**

For all the combinations of IRT types (1PL-IRT or 2PL-IRT) and medical data types, the mean and standard deviation of the estimated latent parameters were in good agreement between pystan and numpyro. In most cases, the sampling time using the Markov chain Monte Carlo method was shorter in numpyro than that in pystan. When the large-sized simulation data were used, numpyro with a graphics processing unit was useful for reducing the sampling time.

**Conclusion:**

Numpyro and pystan were useful for applying the Bayesian 1PL-IRT and 2PL-IRT. Our results show that the two libraries yielded similar estimation result and that regarding to sampling time, the fastest libraries differed based on the dataset size.

## Introduction

Item response theory (IRT) is a statistical framework used for analyzing test results and evaluating test items and test takers quantitatively. While IRT is commonly used in educational and psychological research (Embretson & Reise, 2000; Hays, Morales & Reise, 2000; Cappelleri, Jason Lundy & Hays, 2014), there are several applications of IRT to medical research. For example, Choi et al. (2010) used IRT for constructing the computer adaptive testing system of short-form patient-reported outcome measures with the data from the Patient-Reported Outcomes Measurement Information System project. Gershon et al. (2012) used IRT to build the quality of life item banks for adults with neurological disorders. The most notable example of computer adaptive testing system using IRT is the National Institutes of Health Patient-Reported Outcomes Measurement Information System (PROMIS) (https://www.healthmeasures.net/explore-measurement-systems/promis). PROMIS is an NIH-funded initiative to develop and validate patient reported outcomes for clinical research and practice.

Generally, IRT is applied to the results of binary responses to the test items (*e.g.*, correct and incorrect answers). In medical diagnosis, the results of various diagnostic procedures are frequently defined as binary responses. Therefore, it is possible to apply IRT to the data of medical diagnosis. To apply IRT to the data of medical diagnosis, the following correspondence is assumed: (i) the patient as the test item, (ii) the doctor as the test taker, and (iii) the results of the binary responses obtained through medical diagnosis as test results. For example, Nishio et al. (2020) used IRT for analyzing the results of medical diagnoses by radiologists.

The Bayesian IRT can be implemented using probabilistic programming languages or dedicated libraries (*e.g.*, JAGS, Stan, pystan, and numpyro) (Python Software Foundation, 2023; Depaoli, Clifton & Cobb, 2016; Carpenter et al., 2017; Phan, Pradhan & Jankowiak, 2019). For example, previous studies used Stan for the implementation of the Bayesian IRT, graded response model, and nominal response model (Luo & Jiao, 2017; Nishio et al., 2020; Nishio et al., 2022). The recent advances in hardware and software make it possible to use the Bayesian IRT efficiently. However, there is no study comparing the efficiency of the Bayesian IRT from the viewpoint of computational cost.

The purpose of the current study was to compare the results of the Bayesian IRT implemented with two dedicated libraries (pystan and numpyro). In the current study, the Bayesian 1PL-IRT and 2PL-IRT implemented with pystan and numpyro were applied to the two types of medical data obtained from Nishio et al. (2020). Our main contributions in this article are as follows; (i) the two IRT models could be fitted with pystan and numpyro, (ii) the two libraries yielded similar estimation results for the combinations of the two IRT models and the two types of medical data, and (iii) depending on the dataset size, we evaluated which package had better Markov chain Monte Carlo sampling performance. For reproducibility, our implementation of the Bayesian IRT in pystan and numpyro used in the current study is disclosed as open source through GitHub (https://github.com/jurader/irt_pystan_numpyro). Selections of this article were previously published as a preprint (Nishio et al., 2023).

## Materials & Methods

Because this study used the medical data obtained from Nishio et al. (2020), institutional review board approval or informed consent of patients was not necessary.

### Medical data

The two types of medical data (BONE and BRAIN data) were obtained from Nishio et al. (2020). Table 1 shows the characteristics of the two types of medical data. The BONE data include binary responses from 60 patients (test items) and seven radiologists (test takers), and the BRAIN data include those from 42 patients and 14 radiologists. The total numbers of the binary responses were 420 and 588 in the BONE and BRAIN data, respectively. While the data from two modalities (computed tomography and temporal subtraction) were used in Nishio et al. (2020), those from one modality (computed tomography) were used in this study. As a result, the total numbers of the binary responses were half in this study, compared with Nishio et al. (2020).

**Table 1:** Characteristics of two types of medical data.

Name of data	Number of test takers	Number of test items	Total number of binary responses
BONE data	7	60	420
BRAIN data	14	42	588

### 1PL-IRT

IRT is a statistical model for analyzing the results of binary responses. While there are several types of IRT models (Gelman et al., 2013), 1PL-IRT and 2PL-IRT were used. In the current study, latent parameters of IRT are estimated based on the results of medical diagnoses by test takers.

In 1PL-IRT, one latent parameter (*β*_*i*_) is used to represent the difficulty of test item *i*, and another latent parameter (*θ*_*j*_) is used to represent the ability of test taker *j*. The following equations represent 1PL-IRT.

\begin{eqnarray*}\Pr\nolimits \left( {r}_{ij}=1 \right) & = \frac{1}{1+\exp \nolimits (-{z}_{ij})} \end{eqnarray*}

\begin{eqnarray*}{z}_{ij}& ={\theta }_{j}-{\beta }_{i} \end{eqnarray*}

Here,

• $\Pr \left( {r}_{ij}=1 \right) $ represents the probability that the response of test taker *j* to test item *i* is correct,
• *β*_*i*_ is the difficulty parameter of test item *i*,
• *θ*_*j*_ is the ability parameter of test taker *j*.

### 2PL-IRT

In 2PL-IRT, two latent parameters (*α*_*i*_ and *β*_*i*_) are used to represent test item *i*. The following equations represent 2PL-IRT.

\begin{eqnarray*}\Pr\nolimits \left( {r}_{ij}=1 \right) & = \frac{1}{1+\exp \nolimits (-{z}_{ij})} \end{eqnarray*}

\begin{eqnarray*}{z}_{ij}& ={\alpha }_{i}({\theta }_{j}-{\beta }_{i}). \end{eqnarray*}

Here,

• *α*_*i*_ and *β*_*i*_ are the discrimination and difficulty parameters of test item *i*.

### Experiments

We mainly used Google Colaboratory to run the experiments. The following software packages were used on Google Colaboratory: pystan, version 3.3.0; jax, version 0.4.4; jaxlib, version 0.4.4+cuda11.cudnn82; numpyro, version 0.10.1. Two cores of Intel(R) Xeon(R) (2.20 GHz) and NVIDIA(R) Tesla T4(R) were used as CPU and a graphics processing unit (GPU), respectively.

### Experiments for agreement of latent parameters

The Bayesian 1PL-IRT and 2PL-IRT, implemented with pystan and numpyro, were applied to the BONE and BRAIN data. For 1PL-IRT, the following prior distributions were used:

• *β*_*i*_ ∼ *N*(0, 2),
• *θ*_*j*_ ∼ *N*(0, 2),

where *N* represents a normal distribution in which the first and second arguments are the average and variance of normal distribution, respectively. For 2PL-IRT, the following prior distribution was used in addition to those of 1PL-IRT:

• *log*(*α*_*i*_) ∼ *N*(0.5, 1).

The same prior distributions of the latent parameters were used in both pystan and numpyro.

The following parameters were used for sampling using Markov chain Monte Carlo method in both pytan and numpyro: number of chains (num_chains) = 6, number of samples per one chain (num_samples) = 8,000, number of samples for warmup (num_warmup) = 2,000. For numpyro GPU version, chain_method = ‘parallel’ was used. After the sampling, the posterior distributions of the latent parameters were obtained for the Bayesian 1PL-IRT and 2PL-IRT. The posterior distributions of the latent parameters were then compared between pystan and numpyro.

### Experiments for computational cost

To evaluate the computation cost of 1PL-IRT and 2PL-IRT implemented with pystan and numpyro, simulation data were generated from the medical data (BONE and BRAIN data) and numpyro. The computer simulation was performed in the following steps: (i) estimating the posterior distributions of the latent parameters of 1PL-IRT and 2PL-IRT for the two types of medical data, and (ii) generating binary responses from the IRT equations and the estimated posterior distributions for the two types of medical data. The total number of binary responses in the simulation data were as follows: 420, 840, 2,100, 4,200, 8,400, 21,000, 42,000, 84,000, 210,000, and 420,000 for the BONE data; 588, 1,176, 2,940, 5,880, 11,760, 29,400, 58,800, 117,600, 294,000, and 588,000 for the BRAIN data. This means that size of the simulation data ranged from the original size to 1,000 times. To evaluate the computational cost in pystan and numpyro, the sampling time using Markov chain Monte Carlo method was measured. In numpyro, both CPU version and GPU version were used for the sampling. The following parameters were used for the sampling in both pytan and numpyro: number of chains (num_chains) = 2, number of samples per one chain (num_samples) = 3,000, number of samples for warmup (num_warmup) = 500. For numpyro GPU version, chain_method = ‘parallel’ was used. Due to the limitation of Google Colaboratory, it was not possible to evaluate the sampling time in several large-sized simulation data. Therefore, in addition to Google Colaboratory, we performed the same experiment on a local workstation with Intel Core i9-9820X CPU and Nvidia Quadro RTX 8000.

## Results

### Results for agreement of latent parameters

**Figure 1:**
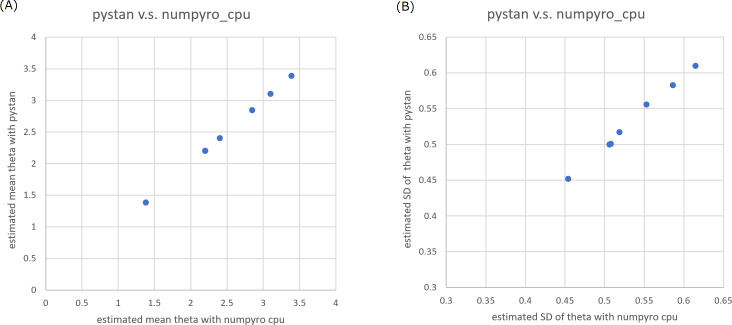
Representative scatter plots of estimated ability parameters of 1PL-IRT between numpyro and pystan for BONE data. (A) Plot for mean of estimated ability parameters, (B) plot for SD of estimated ability parameters. Note: (A) and (B) are obtained from values from Table 2.

**Figure 2:**
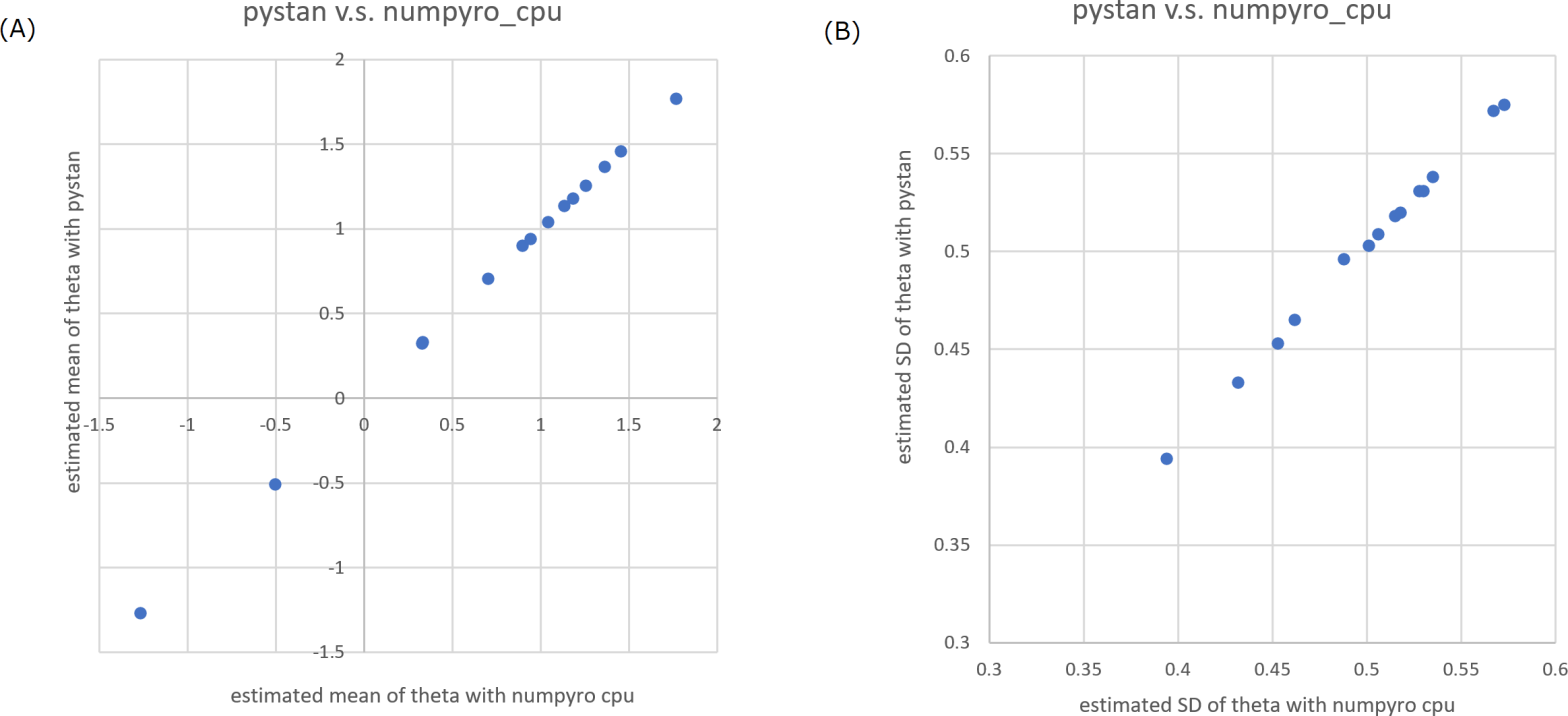
Representative scatter plots of estimated ability parameters of 2PL-IRT between numpyro and pystan for BRAIN data. (A) Plot for mean of estimated ability parameters, (B) plot for SD of estimated ability parameters. Note: (A) and (B) are obtained from values from Table 5.

In the current study, we focused on the ability parameters of test takers, and the estimation results of test items were omitted. Tables 2–5 present the estimation results of the ability parameters of test takers. In addition, Figs. 1 and 2 show representative scatter plots of the estimation results between pytan and numpyro, which are obtained from values of Tables 2 and 5, respectively. Tables 2–5 show the mean, standard deviation, and credible interval (highest density interval) as the estimation results of the ability parameters of test takers. Based on Tables 2–5 and Figs. 1 and 2, we found that there was good agreement between pystan and numpyro for 1PL-IRT and 2PL-IRT of the BONE and BRAIN data.

**Table 2:** Estimation results of ability parameters of test takers in 1PL-IRT for BONE data.

pystan		Estimation				Diagnostics		
		mean	sd	hdi_3%	hdi_97%	ess_bulk	ess_tail	r_hat
	theta[0]	2.201	0.501	1.257	3.133	40373	36621	1
	theta[1]	3.387	0.61	2.25	4.531	47056	35326	1
	theta[2]	3.105	0.583	2.055	4.233	44783	36038	1
	theta[3]	2.845	0.556	1.8	3.882	44444	36888	1
	theta[4]	1.383	0.452	0.511	2.205	38698	37129	1
	theta[5]	2.401	0.517	1.443	3.378	40061	36388	1
	theta[6]	2.2	0.5	1.273	3.153	40777	38464	1
numpyro CPU		Estimation				Diagnostics		
		mean	sd	hdi_3%	hdi_97%	ess_bulk	ess_tail	r_hat
	theta[0]	2.2	0.508	1.277	3.185	32921	34003	1
	theta[1]	3.391	0.615	2.26	4.567	34162	35373	1
	theta[2]	3.099	0.586	2	4.201	35639	34188	1
	theta[3]	2.845	0.553	1.818	3.888	34989	35124	1
	theta[4]	1.382	0.454	0.517	2.225	29675	34343	1
	theta[5]	2.4	0.519	1.449	3.392	33463	34351	1
	theta[6]	2.198	0.506	1.267	3.174	33284	35760	1
numpyro GPU		Estimation				Diagnostics		
		mean	sd	hdi_3%	hdi_97%	ess_bulk	ess_tail	r_hat
	theta[0]	2.199	0.506	1.242	3.134	32251	34882	1
	theta[1]	3.384	0.615	2.252	4.556	34198	36149	1
	theta[2]	3.1	0.585	2.015	4.212	36366	35577	1
	theta[3]	2.843	0.556	1.786	3.875	34938	33051	1
	theta[4]	1.379	0.451	0.538	2.236	30942	34819	1
	theta[5]	2.4	0.521	1.466	3.419	32772	34828	1
	theta[6]	2.199	0.506	1.262	3.163	32300	36210	1

**Notes.**

a Note: theta[i] of Table means *θ*_*i*_ of the equation of 1PL-IRT.

**Table 3:** Estimation results of ability parameters of test takers in 2PL-IRT for BONE data.

pystan		Estimation				Diagnostics		
		mean	sd	hdi_3%	hdi_97%	ess_bulk	ess_tail	r_hat
	theta[0]	2.062	0.49	1.128	2.976	14702	24025	1
	theta[1]	3.086	0.605	1.966	4.231	17011	25730	1
	theta[2]	2.677	0.539	1.664	3.687	16745	26368	1
	theta[3]	2.585	0.571	1.534	3.658	16199	25432	1
	theta[4]	1.252	0.412	0.482	2.029	14322	22764	1
	theta[5]	2.096	0.536	1.102	3.111	13843	23047	1
	theta[6]	2.169	0.511	1.206	3.125	15188	26449	1
numpyro CPU		Estimation				Diagnostics		
		mean	sd	hdi_3%	hdi_97%	ess_bulk	ess_tail	r_hat
	theta[0]	2.057	0.486	1.159	2.991	15715	25398	1
	theta[1]	3.086	0.607	1.943	4.196	17702	27769	1
	theta[2]	2.676	0.546	1.679	3.723	16704	26124	1
	theta[3]	2.581	0.576	1.515	3.674	15881	25012	1
	theta[4]	1.248	0.409	0.494	2.037	15187	26085	1
	theta[5]	2.095	0.533	1.106	3.094	15093	24705	1
	theta[6]	2.167	0.511	1.194	3.104	15545	27676	1
numpyro GPU		Estimation				Diagnostics		
		mean	sd	hdi_3%	hdi_97%	ess_bulk	ess_tail	r_hat
	theta[0]	2.063	0.487	1.159	2.988	14397	25263	1
	theta[1]	3.09	0.601	1.975	4.226	16891	27636	1
	theta[2]	2.68	0.546	1.658	3.701	15578	27217	1
	theta[3]	2.585	0.576	1.525	3.682	14965	24243	1
	theta[4]	1.251	0.411	0.503	2.046	14136	23883	1
	theta[5]	2.096	0.538	1.105	3.112	13277	23510	1
	theta[6]	2.171	0.513	1.237	3.162	14117	24946	1

**Notes.**

Note: theta[i] of Table means *θ*_*i*_ of the equation of 2PL-IRT.

**Table 4:** Estimation results of ability parameters of test takers in 1PL-IRT for BRAIN data.

pystan		Estimation				Diagnostics		
		mean	sd	hdi_3%	hdi_97%	ess_bulk	ess_tail	r_hat
	theta[0]	1.615	0.567	0.569	2.695	19440	27845	1
	theta[1]	0.929	0.53	−0.044	1.946	17105	28114	1
	theta[2]	1.617	0.57	0.561	2.698	19394	29146	1
	theta[3]	0.926	0.527	−0.075	1.902	17568	27120	1
	theta[4]	1.139	0.537	0.145	2.163	18614	28549	1
	theta[5]	1.144	0.543	0.117	2.147	16985	28225	1
	theta[6]	−0.641	0.476	−1.557	0.23	14932	25140	1
	theta[7]	−1.251	0.474	−2.144	−0.362	15336	26550	1
	theta[8]	1.615	0.566	0.548	2.677	19382	29906	1
	theta[9]	0.721	0.515	−0.234	1.702	17127	28476	1
	theta[10]	1.615	0.566	0.568	2.693	19343	29784	1
	theta[11]	1.615	0.567	0.568	2.695	19363	27743	1
	theta[12]	0.166	0.492	−0.766	1.078	15641	26534	1
	theta[13]	0.346	0.499	−0.607	1.264	16040	27328	1
numpyro CPU		Estimation				Diagnostics		
		mean	sd	hdi_3%	hdi_97%	ess_bulk	ess_tail	r_hat
	theta[0]	1.615	0.562	0.613	2.722	18474	28342	1
	theta[1]	0.921	0.527	−0.056	1.924	17348	27983	1
	theta[2]	1.612	0.565	0.545	2.667	19804	27694	1
	theta[3]	0.921	0.527	−0.062	1.922	17238	27787	1
	theta[4]	1.141	0.539	0.122	2.139	17777	28747	1
	theta[5]	1.136	0.538	0.129	2.153	17642	27878	1
	theta[6]	−0.643	0.473	−1.51	0.256	15441	24593	1
	theta[7]	−1.253	0.474	−2.141	−0.36	15186	24014	1
	theta[8]	1.614	0.567	0.555	2.678	19272	27497	1
	theta[9]	0.718	0.515	−0.253	1.69	17100	26777	1
	theta[10]	1.611	0.566	0.519	2.648	19627	29707	1
	theta[11]	1.612	0.568	0.556	2.701	19678	28018	1
	theta[12]	0.163	0.491	−0.752	1.093	15522	25928	1
	theta[13]	0.339	0.499	−0.584	1.294	15960	25830	1
numpyro GPU		Estimation				Diagnostics		
		mean	sd	hdi_3%	hdi_97%	ess_bulk	ess_tail	r_hat
	theta[0]	1.618	0.561	0.581	2.689	18841	29675	1
	theta[1]	0.925	0.527	−0.06	1.914	17399	26758	1
	theta[2]	1.615	0.568	0.564	2.697	19508	27450	1
	theta[3]	0.923	0.527	−0.057	1.929	17223	27357	1
	theta[4]	1.143	0.537	0.151	2.166	17784	29717	1
	theta[5]	1.139	0.541	0.157	2.196	17700	28834	1
	theta[6]	−0.641	0.474	−1.524	0.247	15313	25672	1
	theta[7]	−1.252	0.476	−2.139	−0.354	15033	26341	1
	theta[8]	1.617	0.567	0.551	2.672	19673	26825	1
	theta[9]	0.722	0.515	−0.275	1.67	17220	26307	1
	theta[10]	1.613	0.566	0.559	2.689	19741	28897	1
	theta[11]	1.614	0.566	0.546	2.681	19503	27841	1
	theta[12]	0.166	0.492	−0.754	1.097	15657	26378	1
	theta[13]	0.342	0.498	−0.576	1.307	15644	25302	1

**Notes.**

Note: theta[i] of Table means *θ*_*i*_ of the equation of 1PL-IRT.

**Table 5:** Estimation results of ability parameters of test takers in 2PL-IRT for BRAIN data.

pystan		Estimation				Diagnostics		
		mean	sd	hdi_3%	hdi_97%	ess_bulk	ess_tail	r_hat
	theta[0]	1.18	0.531	0.203	2.181	17825	28333	1
	theta[1]	0.902	0.496	−0.008	1.843	15625	27710	1
	theta[2]	1.254	0.518	0.296	2.235	18845	29397	1
	theta[3]	0.704	0.538	−0.3	1.707	14418	26881	1
	theta[4]	1.136	0.503	0.226	2.111	17740	28371	1
	theta[5]	1.04	0.52	0.096	2.035	17265	28069	1
	theta[6]	−0.509	0.394	−1.235	0.242	12631	24324	1
	theta[7]	−1.27	0.433	−2.075	−0.446	15054	26335	1
	theta[8]	1.458	0.572	0.369	2.512	17362	28080	1
	theta[9]	0.94	0.531	−0.024	1.965	15271	26223	1
	theta[10]	1.767	0.575	0.696	2.852	18638	29705	1
	theta[11]	1.365	0.509	0.411	2.317	18758	29696	1
	theta[12]	0.332	0.453	−0.51	1.193	13643	24442	1
	theta[13]	0.324	0.465	−0.542	1.209	14035	24973	1
numpyro CPU		Estimation				Diagnostics		
		mean	sd	hdi_3%	hdi_97%	ess_bulk	ess_tail	r_hat
	theta[0]	1.183	0.528	0.182	2.157	18389	29763	1
	theta[1]	0.898	0.488	0.009	1.837	16627	28545	1
	theta[2]	1.256	0.515	0.288	2.225	18436	28429	1
	theta[3]	0.704	0.535	−0.291	1.711	14966	25748	1
	theta[4]	1.134	0.501	0.19	2.071	17305	28056	1
	theta[5]	1.044	0.518	0.089	2.031	16558	26045	1
	theta[6]	−0.505	0.394	−1.225	0.262	12487	23078	1
	theta[7]	−1.265	0.432	−2.078	−0.465	15003	25697	1
	theta[8]	1.455	0.567	0.419	2.539	18819	28511	1
	theta[9]	0.943	0.53	−0.035	1.942	16732	27505	1
	theta[10]	1.766	0.573	0.644	2.801	17756	27724	1
	theta[11]	1.365	0.506	0.426	2.326	19432	29606	1
	theta[12]	0.334	0.453	−0.51	1.197	12867	24284	1
	theta[13]	0.327	0.462	−0.523	1.204	13323	26250	1
numpyro GPU		Estimation				Diagnostics		
		mean	sd	hdi_3%	hdi_97%	ess_bulk	ess_tail	r_hat
	theta[0]	1.186	0.527	0.226	2.197	17302	20916	1
	theta[1]	0.897	0.488	0.001	1.827	16923	26620	1
	theta[2]	1.251	0.517	0.292	2.22	15879	16665	1
	theta[3]	0.702	0.532	−0.303	1.695	16956	26275	1
	theta[4]	1.133	0.5	0.204	2.08	18464	28238	1
	theta[5]	1.04	0.515	0.081	2.007	16628	28830	1
	theta[6]	−0.507	0.392	−1.248	0.228	13277	24086	1
	theta[7]	−1.264	0.433	−2.076	−0.458	14157	19916	1
	theta[8]	1.455	0.565	0.395	2.504	17398	23571	1
	theta[9]	0.942	0.528	−0.051	1.925	17510	22661	1
	theta[10]	1.762	0.573	0.644	2.807	16888	24176	1
	theta[11]	1.363	0.504	0.414	2.302	17175	12944	1
	theta[12]	0.33	0.449	−0.503	1.18	13347	25251	1
	theta[13]	0.33	0.463	−0.529	1.203	13968	25446	1

**Notes.**

Note: theta[i] of Table means *θ*_*i*_ of the equation of 2PL-IRT.

From Tables 2–5, Lin’s concordance correlation coefficients (CCC) (Lin, 1989) of the estimated mean of the ability parameters were calculated between (a) pystan *v.s.* numpyro CPU version, (b) pystan *v.s.* numpyro GPU version, and (c) numpyro CPU version *v.s.* numpyro GPU version. The results of CCC values are summarized in Table 6. The following criteria were used to evaluate CCC (Nishio et al., 2016; Kojita et al., 2021); low CCC values (<0.900) were considered to represent poor agreement, whereas higher CCC values represented moderate (0.900–0.950), substantial (0.951–0.990), and almost perfect agreement (>0.990). As shown in Table 6, the CCC values of the estimated mean of the ability parameters indicate almost perfect agreement for 1PL-IRT and 2PL-IRT of the BONE and BRAIN data.

In addition, when the number of samples were fewer (number of samples per one chain was less than 8,000), the agreement of the ability parameters was investigated. The results are shown in Table 7.

**Table 6:** Agreement of estimated ability parameters between pystan *vs.* numpyro CPU version, pystan *vs.* numpyro GPU version, and numpyro CPU version *vs.* numpyro GPU version.

Data type	IRT type	CCC between pystan *vs.* numpyro CPU version	CCC between pystan *vs*. numpyro GPU version	CCC between numpyro CPU version *vs.* numpyro GPU version
BONE data	1PL-IRT	1.000	1.000	1.000
BONE data	2PL-IRT	1.000	1.000	1.000
BRAIN data	1PL-IRT	1.000	1.000	1.000
BRAIN data	2PL-IRT	1.000	1.000	1.000

**Notes.**

Note: The CCC values indicate almost perfect agreement. Because of the significant digits in the CCC calculation, “1.000” was used in Table 6. However, this “1.000” means that the CCC value was approximately 1. Actually, the CCC value of “1.000” was less than 1 (*e.g.*, 0.9999847).

Abbreviation: Lin’s concordance correlation coefficients, CCC.

**Table 7:** Agreement of estimated ability parameters between pystan *vs* numpyro_cpu, pystan *vs* numpyro_gpu, and numpyro_cpu *vs* numpyro_gpu in fewer samples.

Data type	IRT type	Number of samples per one chain	CCC between pystan and numpyro_cpu	CCC between pystan and numpyro_gpu	CCC between numpyro_cpu and numpyro_gpu
BONE data	1PL-IRT	10	0.986	0.994	0.995
BONE data	1PL-IRT	20	0.986	0.998	0.992
BONE data	1PL-IRT	40	0.992	0.999	0.994
BONE data	1PL-IRT	80	0.997	0.999	0.998
BONE data	1PL-IRT	160	0.999	1.000	0.999
BONE data	1PL-IRT	320	1.000	1.000	1.000
BONE data	1PL-IRT	640	1.000	1.000	1.000
BONE data	1PL-IRT	1,280	1.000	1.000	1.000
BONE data	1PL-IRT	2,560	1.000	1.000	1.000
BONE data	1PL-IRT	5,120	1.000	1.000	1.000
BONE data	2PL-IRT	10	0.965	0.971	0.998
BONE data	2PL-IRT	20	0.987	0.990	0.998
BONE data	2PL-IRT	40	0.995	0.994	0.999
BONE data	2PL-IRT	80	0.990	0.992	0.999
BONE data	2PL-IRT	160	0.998	0.998	0.999
BONE data	2PL-IRT	320	1.000	1.000	1.000
BONE data	2PL-IRT	640	0.999	1.000	1.000
BONE data	2PL-IRT	1,280	1.000	1.000	1.000
BONE data	2PL-IRT	2,560	1.000	1.000	1.000
BONE data	2PL-IRT	5,120	1.000	1.000	1.000
BRAIN data	1PL-IRT	10	0.983	0.985	0.999
BRAIN data	1PL-IRT	20	0.994	0.990	0.999
BRAIN data	1PL-IRT	40	0.999	0.999	0.999
BRAIN data	1PL-IRT	80	1.000	0.999	1.000
BRAIN data	1PL-IRT	160	1.000	1.000	1.000
BRAIN data	1PL-IRT	320	1.000	1.000	1.000
BRAIN data	1PL-IRT	640	1.000	1.000	1.000
BRAIN data	1PL-IRT	1,280	1.000	1.000	1.000
BRAIN data	1PL-IRT	2,560	1.000	1.000	1.000
BRAIN data	1PL-IRT	5,120	1.000	1.000	1.000
BRAIN data	2PL-IRT	10	0.990	0.996	0.997
BRAIN data	2PL-IRT	20	0.997	0.997	0.999
BRAIN data	2PL-IRT	40	0.999	0.999	1.000
BRAIN data	2PL-IRT	80	0.999	0.999	1.000
BRAIN data	2PL-IRT	160	1.000	1.000	1.000
BRAIN data	2PL-IRT	320	1.000	1.000	1.000
BRAIN data	2PL-IRT	640	1.000	1.000	1.000
BRAIN data	2PL-IRT	1,280	1.000	1.000	1.000
BRAIN data	2PL-IRT	2,560	1.000	1.000	1.000
BRAIN data	2PL-IRT	5,120	1.000	1.000	1.000

**Notes.**

Note: (i) Except for the number of samples per one chain (num_samples), the parameters for sampling using Markov chain Monte Carlo method are the same as those of the main experiment. (ii) In the main experiment, number of samples per one chain is 8000. (iii) Estimated mean of ability parameter was used in calculating CCC. (iv) Rhat values are not always less than 1.10 in this experiment.

Abbreviation: Lin’s concordance correlation coefficients, CCC.

### Results for computational cost

**Figure 3:**
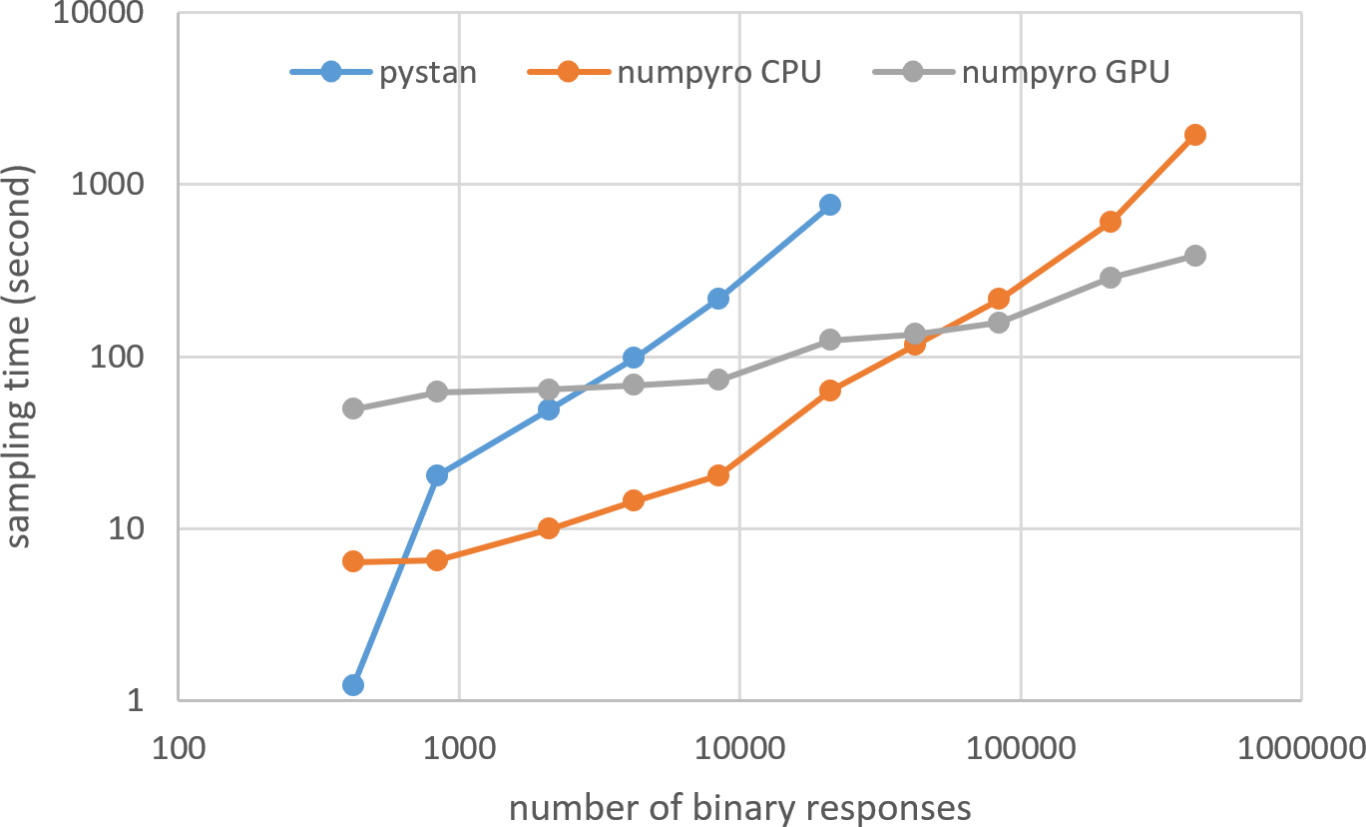
Sampling time of 1PL-IRT for simulation BONE data.

**Figure 4:**
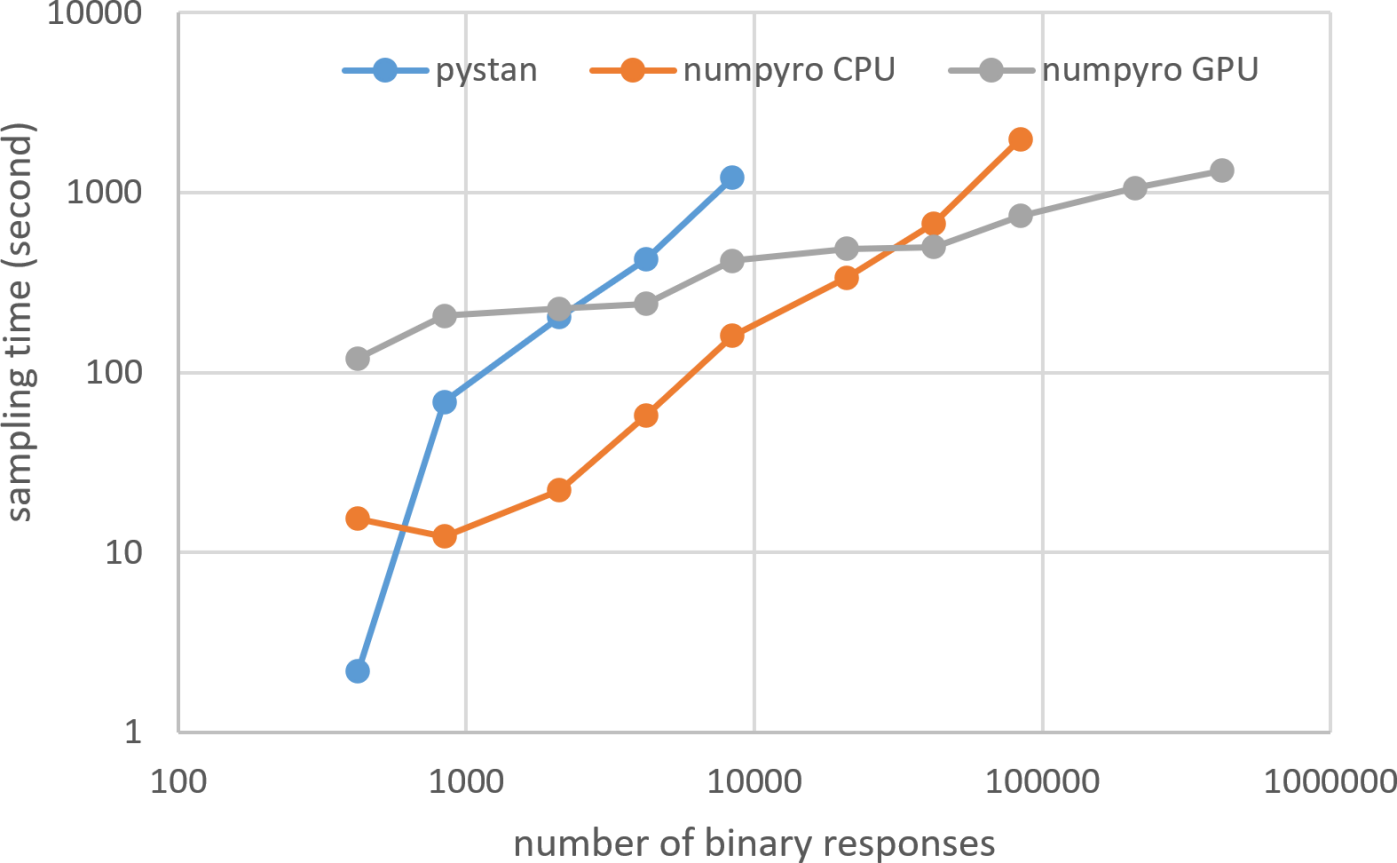
Sampling time of 2PL-IRT for simulation BONE data.

**Figure 5:**
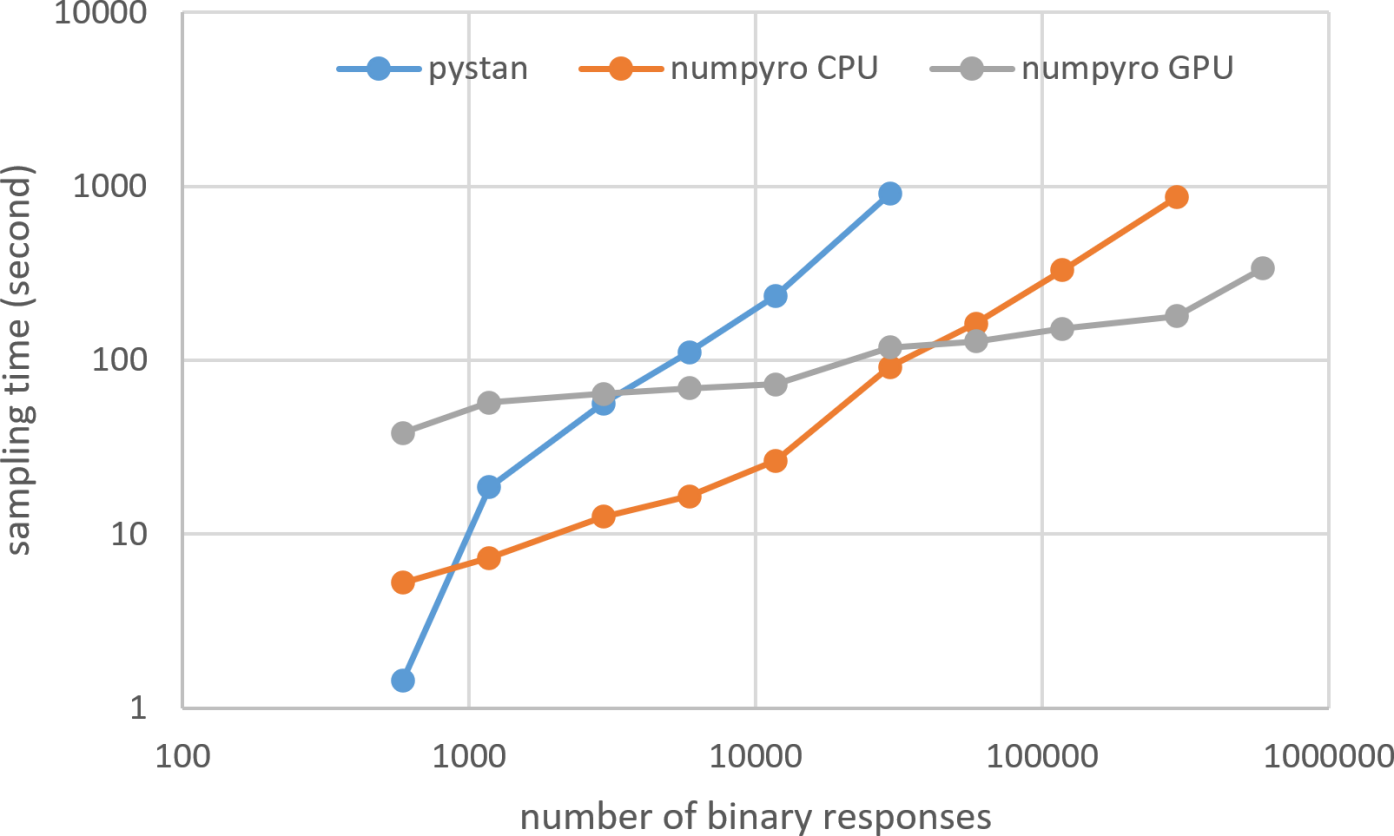
Sampling time of 1PL-IRT for simulation BRAIN data.

**Figure 6:**
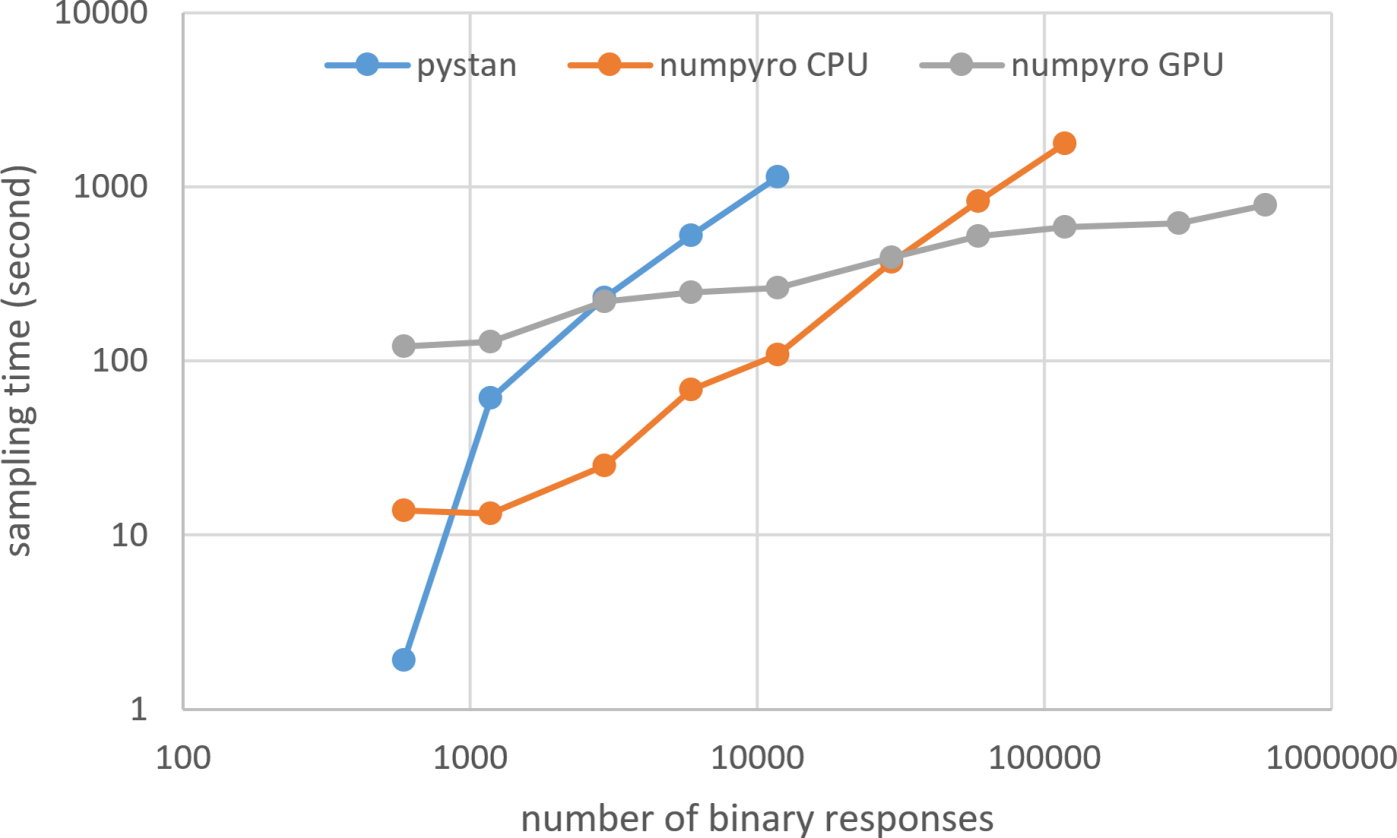
Sampling time of 2PL-IRT for simulation BRAIN data.

**Figure 7:**
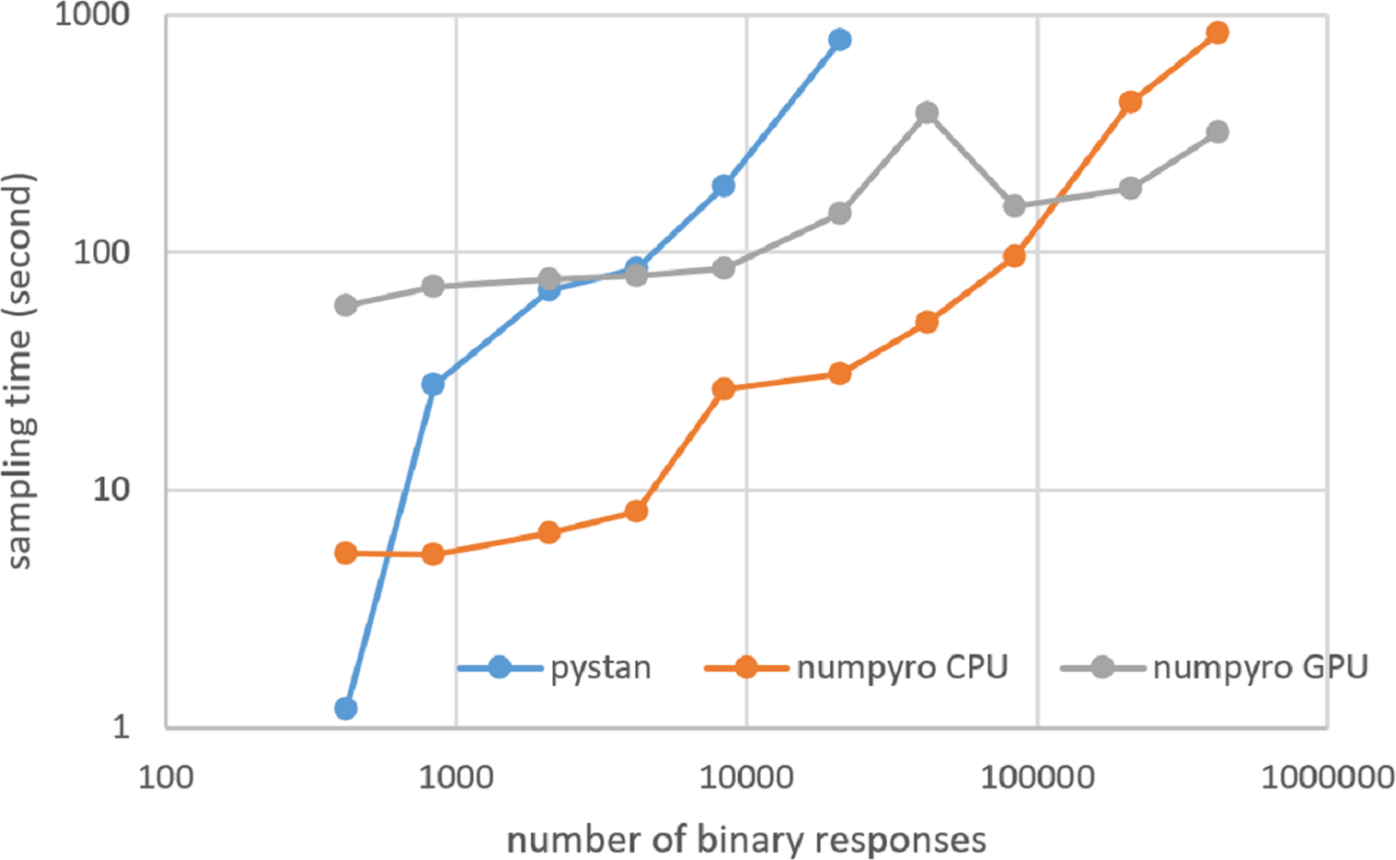
Sampling time of 1PL-IRT for simulation BONE data on local workstation.

**Figure 8:**
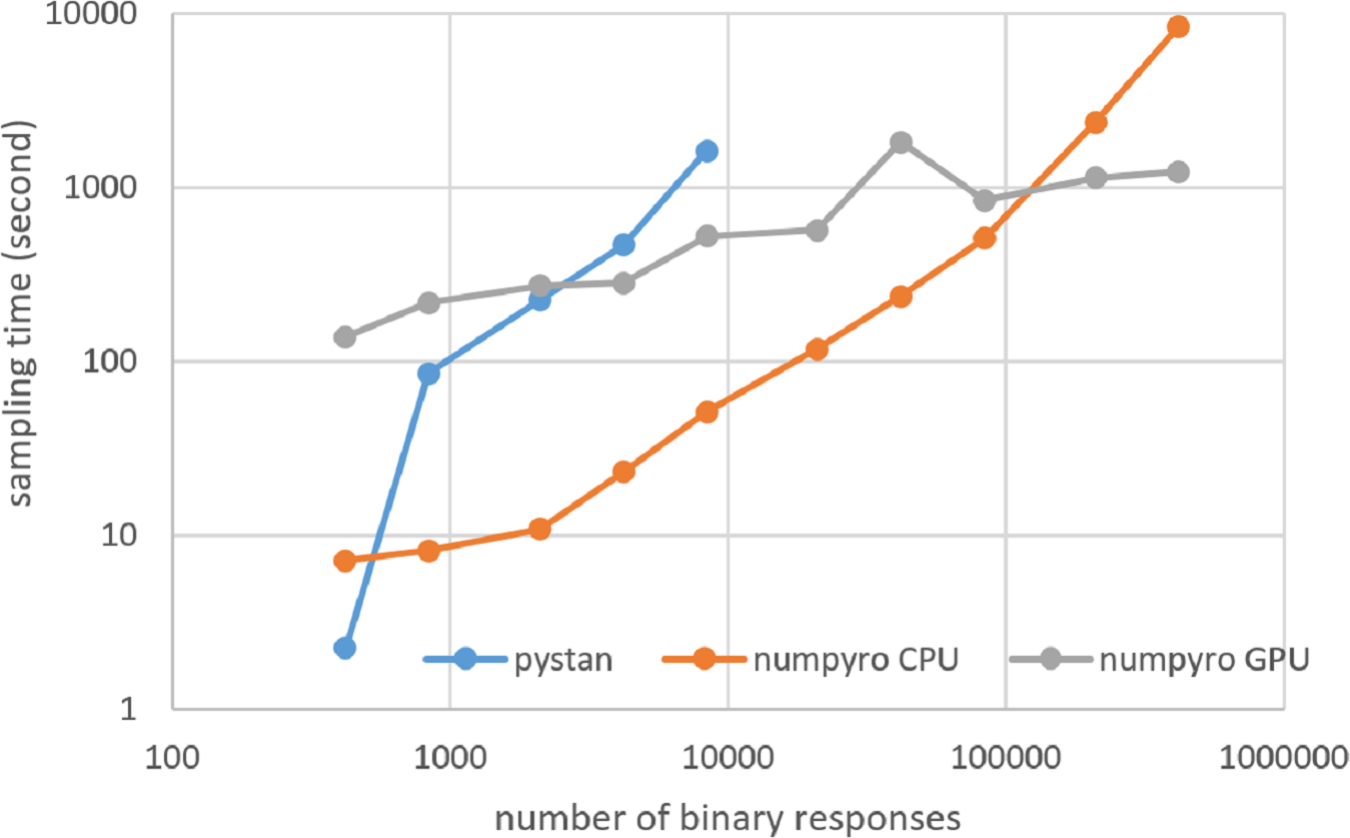
Sampling time of 2PL-IRT for simulation BONE data on local workstation.

**Figure 9:**
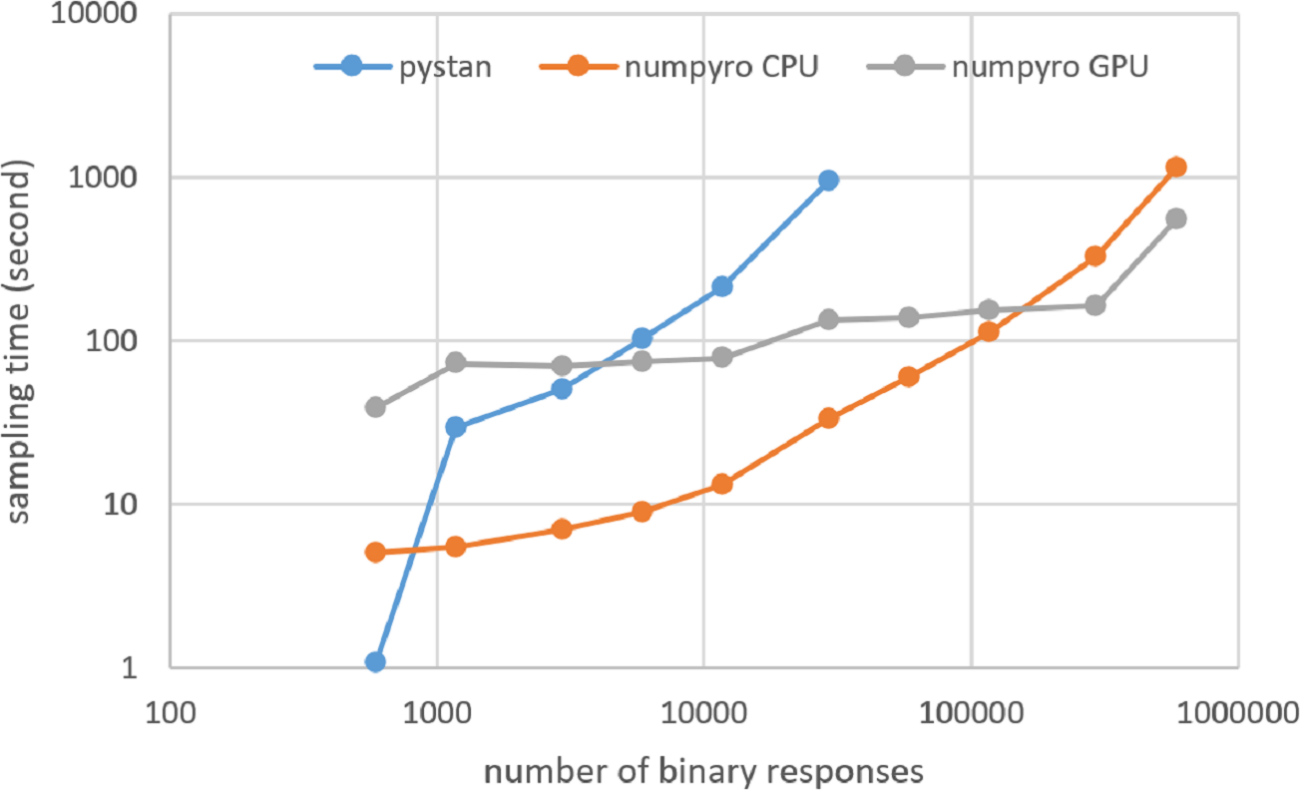
Sampling time of 1PL-IRT for simulation BRAIN data on local workstation.

**Figure 10:**
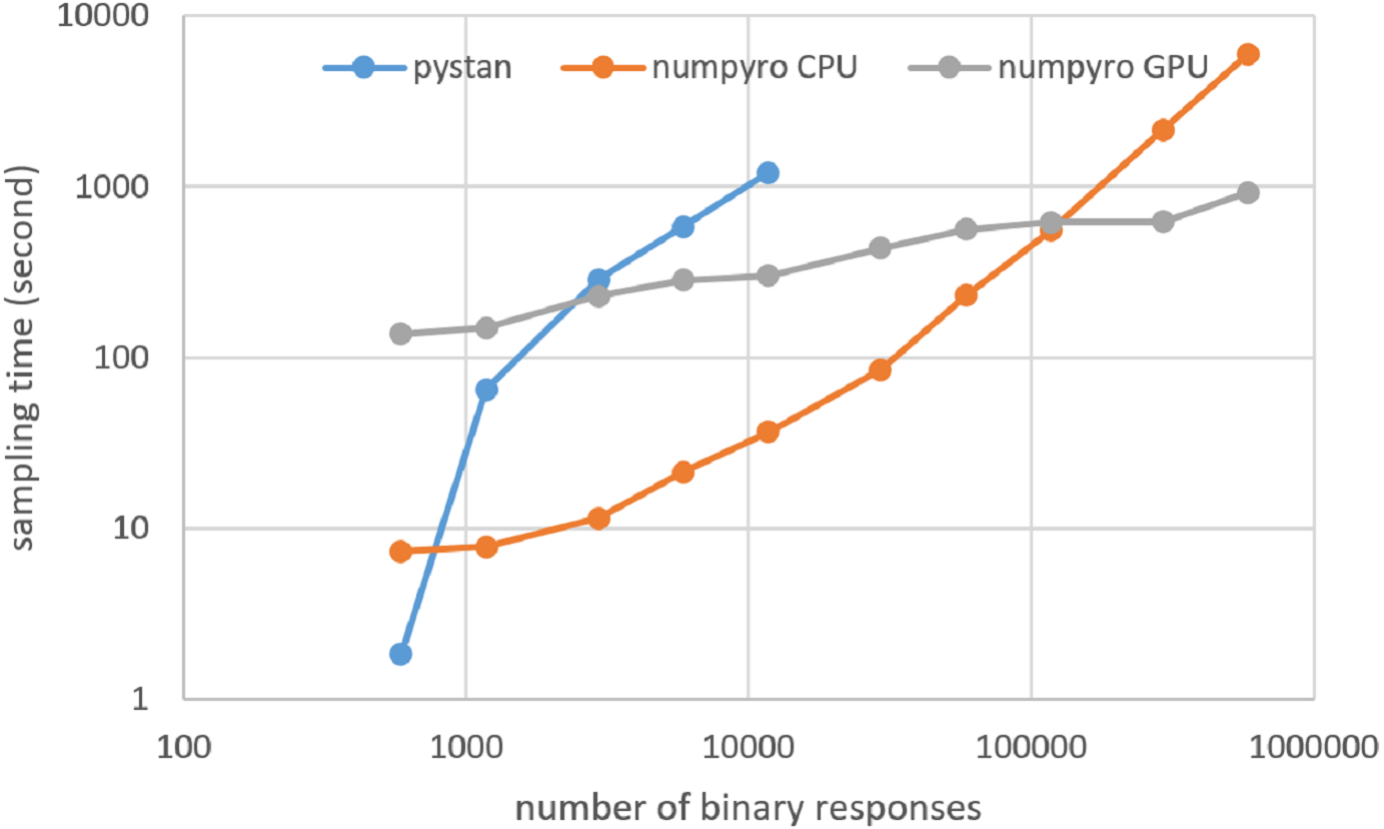
Sampling time of 2PL-IRT for simulation BRAIN data on local workstation.

Figures 3–6 show the sampling time for the simulation data of the BONE and BRAIN data. When original-size simulation data were used, the sampling time was shorter in pystan than numpyro CPU version. However, in the simulation data of the BONE and BRAIN data except for the original size, the sampling time was shorter in numpyro CPU version than pystan. Moreover, when the large-sized simulation data (total number of binary responses > 30,000–50,000) were used, the sampling time was shorter in numpyro GPU version than numpyro CPU version. In addition to the experiments using Google Colaboratory, the results of computational cost on the local workstation are shown in Figs. 7–10. The same trend was observed when using Google Colaboratory and the local workstation.

## Discussion

The current study aimed to compare the estimation results of the ability parameter of test takers using two different libraries (pystan and numpyro) for two different types of IRT models and medical data (BONE and BRAIN data). The study found that there was good agreement between pystan and numpyro for all the combinations of the IRT models and medical data; there was almost perfect agreement between pystan and numpyro in the CCC values of the estimated mean of ability parameters. The current study also compared the sampling time for the simulation data of the BONE and BRAIN data. We found that while the sampling time was shorter in pystan than numpyro CPU version for the original-size data, it was shorter in numpyro CPU version than pystan for the simulation data except for the original size. For the large-sized simulation data, the sampling time was shorter in numpyro GPU version than numpyro CPU version.

Our results show that there was almost perfect agreement in the ability parameters of the Bayesian IRT between pystan and numpyro. This suggests that researchers can choose either library for implementing the Bayesian IRT. While numpyro requires only Python, both Python and Stan (two different programming languages) are necessary for pystan. Many practitioners and researchers may find numpyro to be simple and straightforward.

**Figure 11:**
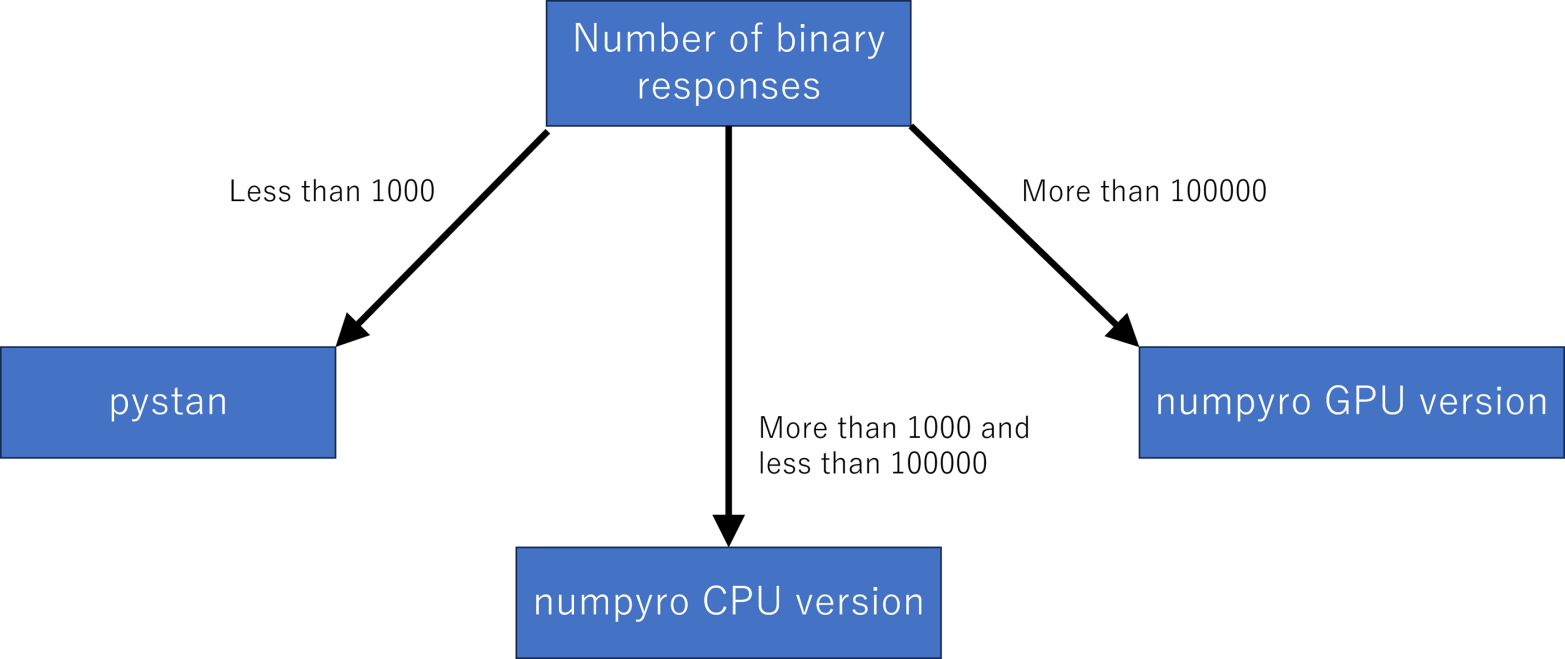
Recommendation for selecting pystan and numpyro.

Although we used the simulation data, our results of sampling time show that the fastest libraries differed based on the total number of binary responses. Specifically, pystan was the fastest for the original-size simulation data, while numpyro CPU version was the fastest for the small-sized and medium-sized data. For the large-sized simulation data, the sampling time was shorter in numpyro GPU version than numpyro CPU version. This implies that practitioners and researchers should select either pystan or numpyro based on the data size. Figure 11 shows our recommendation for selecting pystan and numpyro.

Tables 3 and 4 show that the total number of latent parameters may affect the usefulness of GPU for reducing the sampling time. In complex models, such as 2PL-IRT used in this study, numpyro GPU version may tend to be faster than numpyro CPU version. The effect of GPU on the sampling time should be evaluated in future studies.

This study had several limitations. First, we evaluated only Bayesian 1PL-IRT and 2PL-IRT. Further studies are needed to investigate the effectiveness of pystan and numpyro in other types of Bayesian models. Second, although we evaluated the sampling time, we used the simulation data instead of real-world data. Future studies should use real-world data to evaluate the sampling time. Third, we mainly used Google Colaboratory. Although Google Colaboratory has several merits (*e.g.*, ease of use and availability), our experiments were performed using limited types of hardware. Fourth, because the data and purpose of our study are different from those of Nishio et al. (2020), it is impossible to compare our results with those from that article.

## Conclusions

The current study demonstrated that both pystan and numpyro were effective in the estimation for 1PL-IRT and 2PL-IRT of the BONE and BRAIN data. Our results show that the two libraries yielded similar estimation results. In addition, our results of sampling time show that the fastest libraries differed based on the total number of binary responses.

## Additional Information and Declarations

### Data Availability

The following information was supplied regarding data availability: The code is available at GitHub and Zenodo: - https://github.com/jurader/irt_pystan_numpyro
- jurader. (2023). jurader/irt_pystan_numpyro: First release (1st_release). Zenodo. https://doi.org/10.5281/zenodo.8187939
The data is available at: Nishio M, Akasaka T, Sakamoto R, Togashi K. Bayesian Statistical Model of Item Response Theory in Observer Studies of Radiologists. Acad Radiol. 2020 Mar;27(3):e45-e54. doi: 10.1016/j.acra.2019.04.014. Epub 2019 May 28.
